# Impact of Host Genes and Strand Selection on miRNA and miRNA* Expression

**DOI:** 10.1371/journal.pone.0023854

**Published:** 2011-08-31

**Authors:** Marta Biasiolo, Gabriele Sales, Marta Lionetti, Luca Agnelli, Katia Todoerti, Andrea Bisognin, Alessandro Coppe, Chiara Romualdi, Antonino Neri, Stefania Bortoluzzi

**Affiliations:** 1 Department of Biology, University of Padova, Padova, Italy; 2 Matarelli Foundation, Department of Pharmacology, University of Milano, Milano, Italy; 3 Department of Medical Sciences, University of Milano, Hematology 1-CTMO, Foundation IRCCS Ca' Granda, Ospedale Maggiore Policlinico, Milano, Italy; Istituto Dermopatico dell'Immacolata-IRCCS, Italy

## Abstract

Dysregulation of miRNAs expression plays a critical role in the pathogenesis of genetic, multifactorial disorders and in human cancers. We exploited sequence, genomic and expression information to investigate two main aspects of post-transcriptional regulation in miRNA biogenesis, namely strand selection regulation and expression relationships between intragenic miRNAs and host genes. We considered miRNAs expression profiles, measured in five sizeable microarray datasets, including samples from different normal cell types and tissues, as well as different tumours and disease states. First, the study of expression profiles of “sister” miRNA pairs (miRNA/miRNA*, 5′ and 3′ strands of the same hairpin precursor) showed that the strand selection is highly regulated since it shows tissue-/cell-/condition-specific modulation. We used information about the direction and the strength of the strand selection bias to perform an unsupervised cluster analysis for the sample classification evidencing that is able to distinguish among different tissues, and sometimes between normal and malignant cells. Then, considering a minimum expression threshold, in few miRNA pairs only one mature miRNA is always present in all considered cell types, whereas the majority of pairs were concurrently expressed in some cell types and alternatively in others. In a significant fraction of concurrently expressed pairs, the major and the minor forms found at comparable levels may contribute to post-transcriptional gene silencing, possibly in a coordinate way. In the second part of the study, the behaved tendency to co-expression of intragenic miRNAs and their “host” mRNA genes was confuted by expression profiles examination, suggesting that the expression profile of a given host gene can hardly be a good estimator of co-transcribed miRNA(s) for post-transcriptional regulatory networks inference. Our results point out the regulatory importance of post-transcriptional phases of miRNAs biogenesis, reinforcing the role of such layer of miRNA biogenesis in miRNA-based regulation of cell activities.

## Introduction

The discovery of microRNA-based post-transcriptional regulation of gene expression added a novel level of genetic regulation to a wide range of biological processes, including cell differentiation, organogenesis and development [1]–[3]. Dysregulation of miRNAs expression plays a critical role in the pathogenesis of genetic and multifactorial disorders (http://www.mir2disease.org/) and of most, if not all, human cancers [4].

Two different aspects of miRNAs biogenesis have been studied by integration of genomic information with sequence and expression data, specifically i) the strand selection bias, affecting all miRNAs and involving the mature pairs (“sister”) derived from the same hairpin precursors; and ii) the processing of intragenic miRNAs with the corresponding host gene transcripts.

With regard to strand selection bias, two different mature miRNAs sequences can be derived from the same precursor hairpin: a major, the stable and prevalent form, and a minor, the unstable one, degraded. The two forms are associated to different sets of target genes, thus contributing in different ways to the regulation of cell activities; experiments conducted on selected miRNAs pairs demonstrated that they could be both functionally effective [5].

According to the conventional model, Dicer cleaves the pre-miRNA hairpin to produce a miRNA duplex (∼22 nt), which is incorporated into the RISC. The RISC recognizes the duplex, unwinds it, selects the guide miRNA strand (while degrading the passenger strand), and mediates recognition and silencing of target RNAs. To date, such asymmetry of the strand selection process is considered determined by differential thermodynamic stability of alternative sister miRNAs (“strand bias” theory, as in [6], [7]), although additional features possibly acting as miRNA strand selection determinants in humans and flies were also investigated [8]. In contrast, fragmentary but interesting evidences of regulated and tissue-dependent paired expression of sister miRNAs have been reported [5]. To support this, a recent paper has been published reporting sequencing and characterization of bovine miRNAs [9], which underlined that only 60% of them displayed thermodynamic stability-dependent strand selection bias. These studies introduced innovative concepts and unravelled that i) both sister mature miRNAs may be accumulated in some tissues and cell types, and ii) the strand selection might not be deterministic but tissue-specific, so that a given strand could be guide strand in a specific cell type and passenger in another one. The crucial pathogenetic role of the passenger strand has been pointed out by a study on thyroid cancer [10].

Our results on genome-wide investigation of co-expression relationships between mature sister miRNAs highlight that different biological contexts most likely share complex mechanisms of strand selection regulation, leading either to alternative expression of a single mature form or to concurrent expression of both mature sister miRNAs.

Another aspect of miRNAs characteristic is the co-expression of intragenic miRNAs and host genes which is crucial to i) elucidate possible transcriptional and post-transcriptional regulatory circuits and ii) clarify, from a methodological point of view, whether host genes expression data may be profitably used as proxy of intragenic miRNAs expression.

Mature miRNAs can cause translation inhibition or mRNA cleavage, by base pairing with the 3′ untranslated region (3′-UTR) of their target mRNAs, depending on the complementarity degree between the miRNA and its target sequence [11], [12]. Target mRNAs of miRNAs can be predicted by computational methods. Although comparative evaluations of different target prediction methods provided some kind of ranking of the sensitivity of different algorithms [13]–[15], it is well known that any available software produces a large fraction of false positive predictions. This might be due not only to the limited comprehension of the molecular basis and effect of miRNA-target pairing, but also to context dependency of post-transcriptional regulation. Thus, the integration of target predictions with miRNA and target mRNA expression profiles has been proposed to select functional miRNA-mRNA relationships, according to increasing experimental evidences which supported the miRNA mechanism of target degradation rather than translational repression. Since miRNAs tend to down-regulate target mRNAs [16]–[18], the expression profiles of genuinely interacting pairs are expected being anti-correlated. The integrative analysis [19]–[21] allows the selection of plausible in-silico predictions [22], gaining insights into the reconstruction of regulatory networks that govern genetic pathways of important biological processes. The main limitation of such integrative approach is the relative shortage of matched miRNA/mRNA expression datasets (i.e. miRNA and mRNA expression measures in exactly the same set of biological samples).

Recently, some evidences have been provided that in specific contexts some miRNAs are co-expressed with their host genes [23]–[25]. This led to suggestion that miRNA host genes expression profiles might be used as possible proxy for the expression profile of the embedded miRNA [26]. Other reports, as Polster et al. 2010 [27], pointed out specific cases of discordant expression of miRNA and host genes. To clarify if and how much intragenic miRNAs are co-expressed with host genes, we thus collected different human datasets of miRNAs and genes expression profiles in normal tissues and tumour samples and extensively studied the co-expression of intragenic miRNAs and their host genes, to obtain a fairly broad picture of their relationship.

## Results

Table 1 shows details about microarray-based expression datasets considered for each different analysis performed in this study. Datasets were selected to obtain expression profiles of large numbers of known miRNAs, measured in many samples, representing fairly different biological contexts. As detailed in the Materials and Methods section, out four microarray-based datasets including matched miRNA and gene expression profiles, two regard blood cells (Multiple Myeloma and normal plasma cells samples (MM) [20], Acute Lymphoblastic Leukaemia samples (ALL) [28]) whereas the other two regard parietal lobe cortex (normal and with in Alzheimer's disease, ALZ) and prostate (normal and cancer). A fifth dataset, include miRNA only expression profiles in 8 different cancer types and corresponding normal tissues samples (MCN) [29]. The number of miRNAs represented in each expression dataset is also indicated in Table 1.

**Table 1:** Schema of expression datasets used for different levels of analyses in this study.

			DATASETS				
			Matched miRNA and genes expression data				miRNA-only expression data
			MM	ALL	ALZ	PRO	MCN
**Total number of miRNAs in the original series matrix**			722	470	462	373	722
**ANALYSES**	**sister miRNA pairs**		√	√	√	√	√
	**Intragenic miRNA/host gene**	**Co-expression**	√	√	√	√	
		**Real/Proxy for network reconstruction**	√	√	√	√	

Among five expression datasets obtained by microarray technology, four comprise matched miRNA and gene expression, whereas one includes only miRNA expression data.

We considered all mature miRNAs in miRBase, where 676 (corresponding to 869 mature sequences) were assigned to unique genomic locations whereas the remaining were discarded, since unmapped or corresponding to more than one different localization per miRNA.

### Expression of sister mature miRNA pairs belonging to the same hairpin

Two different mature miRNA sequences (miRNA/miRNA*) are generated from a fraction of precursor hairpins and are associated to different sets of target genes and regulated cell activities. We considered expression profiles of mature miRNAs, obtained by microarray platforms specifically designed to measure mature forms.

Part of miRNA hairpin sequences is represented, in each dataset, by two different mature miRNAs, each one associated to an individual expression profile. Identical mature pairs derived from hairpins belonging to different genomic localizations were considered only once, obtaining a set of 237 couples of sister mature miRNAs from the same hairpin represented in the MCN, MM datasets was considered. In ALL, ALZ and PRO datasets we found respectively 32, 37 and 95 sister miRNA pairs.

**Figure 1:**
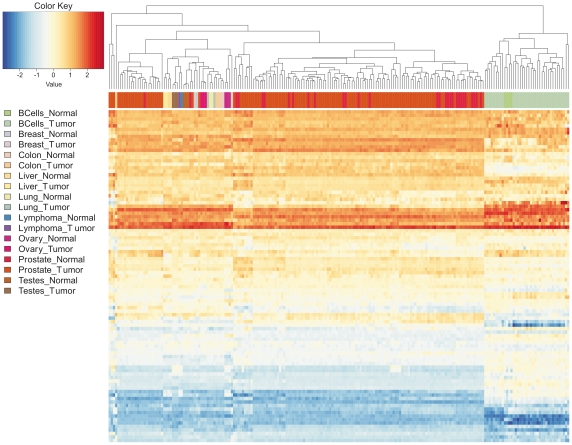
Variability of strand selection bias across samples. The heatmap evidences patterns prevalence for a set of 95 5′/3′ sister miRNA pairs obtained by the combination of three out of five considered datasets giving rise to the maximum number of represented miRNA pairs (MM, PRO and MCN). The group includes 211 samples representing normal and malignant B cells plus two sets of solid tumours and corresponding normal tissues. Lines and columns of the heatmap respectively represent miRNA pairs and samples ordered by hierarchical cluster analysis of standardized per sample expression values log_2_(ratio) of sister miRNA pairs. Samples are tagged according to cell or tissue type and to normal or cancer state, to facilitate the interpretation of sample clustering. The red-blue colour scale indicates the extent of prevalence of one or another miRNA in the pair. A positive (red) value indicates that, in a given sample, the 5′ miRNA is more expressed than the 3′ miRNA, negative (blue) values indicates the opposite case and comparable expression values between sister miRNAs are indicated by log_2_(ratio) values around 0 and are shown in white or pale colours. The heatmap shows clearly the existence of pairs in which only one miRNA is prevalent across the majority of samples, but also pairs showing variable strand selection bias in different sample groups, representing different tissue types. Moreover, sample clustering based on standardized per sample log_2_(ratio) of sister miRNAs expression values is able to fairly well classify different tissues, and in case of MM, to distinguish normal and malignant B cells.

The investigation of expression relationships between sister miRNA pairs provided interesting clues about strand selection bias regulation. We were willing to understand if one miRNA in the pair is more expressed than the other in all tissues/cell types/conditions or if the strand selection bias may be cell/tissue-specific. The cluster analysis of samples and of miRNAs pairs according to standardised per sample log2(ratio) between expression values of 5′ and 3′ sister miRNAs provides a general picture of expression prevalence among sister miRNA pairs. Only five miRNA pairs are represented in all considered datasets (Figure S1). The heatmap in Figure 1 shows patterns of 5′/3′ prevalence for a set of 95 sister miRNAs in 211 samples deriving from the combination of three datasets giving rise to the maximum number of miRNA pairs (MM, PRO and MCN). It is worth notice that heatmaps in Figure 1 and S1 are not quantitative results derived from expression data meta-analysis, but rather they provide qualitative information about the prevalence among sister miRNA pairs.

We found that the standardised log_2_(ratio) of expression values between two sister miRNAs, is able to fairly well separate different samples/tissue types. Even if a laboratory/study effects can not be excluded, normal and malignant B cells, derived from the same dataset (MM) are correctly separated, suggesting that standardised log_2_(ratio) of expression values may help distinguish normal and tumour samples. Figure 1 shows that, for a considerable fraction of the pairs, the same miRNA is the most expressed in the majority of considered samples. No general prevalence of 5′ or 3′ strands was observed. Among pairs expressed at comparable level in part of considered samples, only minority are associated to standardised log_2_(ratio) of expression values close to zero in all considered samples. We can conclude that for the large majority of pairs the strand selection bias may be tissue/cell specific. Indeed, the heatmap shows lines in which positive and negative values are mixed, corresponding to sister miRNA pairs showing a not deterministic strand selection bias. At least two sets of miRNAs seem to be expressed in B cells with inverse ratio respectively to other tissues. For instance, 22 pairs shows mean values of expression log_2_(ratio) in the two sample sets of opposite sign, and 16 miRNA pairs shows mean values of expression log_2_(ratio) in MM and in all the other samples differing at least one point in the scale of standardised values.

**Figure 2:**
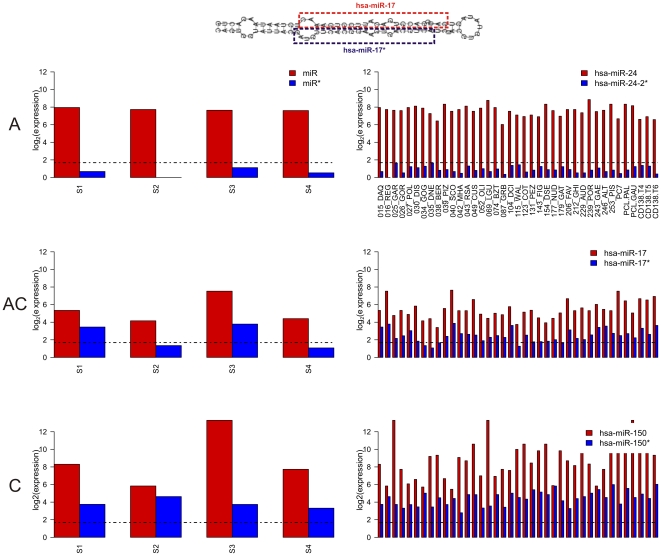
miRNA sister pairs categories. miRNA sister pairs were classified according to their tendency of being concurrently or alternatively expressed in those samples in which at least one of the pairs is expressed over the threshold (median of all expression values). Left panels show the criteria for classification, using example expression profiles in four theoretical samples (S1-4) for a general miRNA pair (miR/miR*). Single miRNAs are considered expressed in those samples with signal intensity over the threshold (black dotted line). A sister pair may result alternatively (A) or concurrently (C) expressed, in a given sample. Then, considering expression in all samples, a sister pair will be: alternatively expressed (A; the two miRNAs of the pair are never expressed together in considered samples); alternatively expressed in some samples and concurrently expressed in others (AC); always concurrently expressed in the same set of samples (C). For each category, right panels show example expression profiles in MM samples of specific miRNA pairs belonging to the category.

Many mature miRNAs are characterized by low expression values, slightly over background, and possibly associated to miRNA cellular concentrations insufficient to guarantee the biological activity. Thus, as explained in Methods, mature miRNAs were tagged as “expressed” in a given sample whenever the expression level was higher than the median of all expression values in the matrix. Then, sister miRNA pairs may be alternatively (i.e. only one out of two sister miRNAs is present) or concurrently expressed (both miRNA and miRNA* are present) in a given sample. Therefore, for each of the five considered datasets, miRNA pairs fall in one of the following categories (Figure 2, Table 2):

A: alternatively expressed, with concurrent expression never occurring in considered samples;
C: concurrently expressed pairs in the same set of samples (expressed concurrently whenever expressed);
AC: miRNAs pairs resulting alternatively expressed in some samples and concurrently expressed in others.

According to microarray data, the majority of miRNA pairs (60%±25%, mean and standard deviation across datasets) belong to the AC class, whereas a more or less small percentage results always concurrently expressed (11%±10%, maximum 27%). Pairs showing pure “alternative” behaviour, according to the classical biogenesis model, represent less than one quarter of total expressed pairs, in average (23%±7%). When two mature forms are expressed in the same sample, we considered the comparability between their expression levels, as per sample expression ratios distribution among C pairs expression levels, in those samples showing concurrent expression. We considered that two expression levels are comparable when their absolute value of expression log_2_(ratio) not exceeds 1. Excluding the PRO dataset, in which only one C pair was recorded, in the remaining datasets in average 17%±13% of miRNA pairs have comparable expression levels. Moreover, the distribution of log_2_(ratio) among expression levels of AC class miRNA pairs shows that about one third of them (34%±28%), are expressed at comparable level.

**Table 2:** miRNA sister pairs classification.

	PRO		MM		ALZ		ALL		MCN	
	#	%	#	%	#	%	#	%	#	%
**C**	1	1.1	13	5.5	10	27	4	12.5	25	10.5
**AC**	75	78.9	197	83.1	7	18.9	19	59.4	144	60.8
**A**	19	20	26	11	10	27	9	28.1	65	27.4
**Total expressed**	95	100	236	99.6	27	73	32	100	234	98.7
**Both not expressed**	0	0	1	0.4	10	27	0	0	3	1.3

Categories of miRNAs pairs derived from the same precursor were classified according to their expression characteristics, for each dataset, in: alternatively expressed (A); concurrently expressed (C) or alternatively expressed in some samples and concurrently expressed in others (AC).

The heatmap in Figure S2 reports, for sister miRNA pairs and datasets considered in Figure 1, patterns of prevalence recalculated according the above reported miRNA pairs classification and considerations.

### miRNA are hosted by long genes

**Figure 3:**
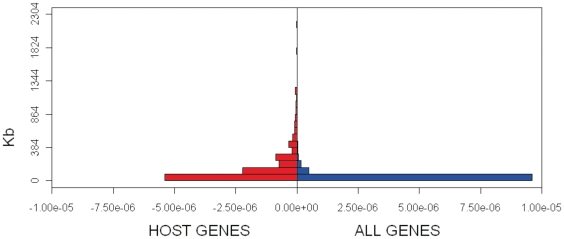
Host genes are relatively long. The back-to-back histogram compares the length distribution of host genes with that of all human genes. Host genes are longer than expected by chance and the difference is highly significant.

A few studies considered miRNAs host genes genomic length/organization and their possible regulatory role. In particular, Golan and colleagues [30] observed that miRNA genes are hosted within introns of short genes and hypothesised that miRNA integration into short genes might be evolutionary favourable due to interaction with the pre-mRNA splicing mechanism. Here, we evaluated the length of the 279 host genes in comparison with all human genes. The average gene span of the 279 host genes (180867 nt; Wilcoxon rank sum test p-value 2.2*10^−16^, Figure 3) is significantly longer (on average 6 times) than that of remaining 49, 506 human genes (29, 945 nt).

### Limited co-expression of intragenic miRNAs and host genes

We considered the pair-wise expression correlations of respectively 309, 147, 148 and 170 mature miRNA/host gene pairs in the MM, ALL, ALZ and PRO datasets (Table 3). In all datasets, more than one half of miRNA/host pairs (63±13, average and standard deviation across datasets) were positively correlated, with slightly positive value for the median correlation per dataset. However using a criterion of FDR<0.01, no pairs meet a correlation significance in the ALZ dataset, whereas in the remaining datasets from the 5% to the 36% of correlations result significant. Overall, our data indicated that in all four different datasets a large majority of miRNA/host gene expression profiles are not significantly positively correlated and are instead poorly correlated or even anti-correlated, in contrast with the notion that intragenic miRNAs are co-expressed with host genes.

**Table 3:** Intragenic miRNAs and host genes correlations.

		MM		ALL		ALZ		PRO	
**miRNA-host gene correlation**	**Total**	309	%	147	%	148	%	170	%
	**Positive**	199	64	81	55	77	52	138	81
	**>0.25**	78	25	53	36	47	31	56	33
	**>0.5**	33	11	20	13	21	14	13	8
	**FDR<0.01**	34	11	8	5	0	0	60	36

The correlation between intragenic miRNA and host genes expression profiles tends to be slightly positive, but with prevalently low percentages of significantly positively correlated pairs.

We reasoned that about 20% considered host genes is associated each to two mature miRNA forms, derived from the same hairpin whereas the remaining host genes are associated to only one mature miRNA. Since sister miRNA expression profiles may not be considered independent, we carried out again, for each of the four expression datasets, the above reported analysis of co-expression between intragenic miRNAs and host genes, but considering, for host genes including two mature miRNAs, only the mature miRNA of the pair with the highest miRNA-host correlation. Considering only the highest miRNA-host gene correlation, when a pre-miRNA hosted in a gene produces two mature forms, may give an overestimation of general miRNA-host co-expression tendency. Anyway, the percentages of miRNA-host correlations being positive, >0.25 and >0.5, for each dataset (data not shown), resulted to be almost equal to that reported in Table 3 and showed limited co-expression of intragenic miRNAs with host genes.

### Impact of host genes expression used as proxy for miRNAs on target selection

These observations discouraged the usage of host gene expression profiles as a proxy to monitor the expression of its embedded miRNA. Thus, we tested whether such procedure affected the results of an integrated analysis of target prediction with miRNA and target expression profiles, using datasets in which real and not inferred miRNAs expression data are available. In particular, for each of the four miRNA and genes matched datasets, a comparative evaluation of results was obtained, by contrasting two integrated analyses, the first (REAL) was conducted on real miRNA and gene expression profiles, whereas the second one (PROXY) was conducted on host genes expression profiles, used as proxy for miRNAs, and gene expression profiles. For each dataset, different numbers of miRNA and genes were considered for target prediction, using TargetScan [31], after filtering out those miRNAs with almost invariable profile (25% with lower Shannon entropy) and/or weakly expressed (25% with lowest average values). For each dataset, both for the REAL and PROXY analysis, the sets of predicted relationships mostly supported by expression profiles anti-correlation analysis were identified according to different percentile cut-offs on miRNA-target expression profiles anti-correlation values [19]–[21], [26], [32]. It is worth notice that in different studies cutoffs around 1–3% were considered adequately stringent for a selection of candidate functional miRNA-target relationships.

**Figure 4:**
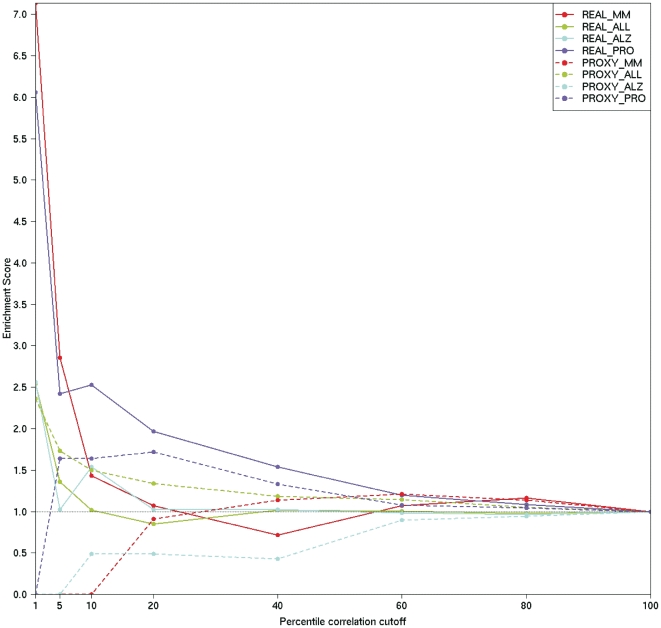
Enrichment in validated miRNA-target relations obtained by REAL and PROXY analyses of different datasets. Comparative evaluation of integrated analysis results was performed using real miRNA and gene expression profiles (REAL) and host genes expression profiles, as proxy for miRNAs, and gene expression profiles (PROXY). For each dataset, first we filtered out miRNAs with almost invariable or weak expression, then we identified the miRNA and genes target prediction set using TargetScan. Both for the REAL and PROXY analysis, the groups of predicted relationships most supported by expression profiles anti-correlation analysis were identified according to different percentile of anti-correlation cut-offs. A subset of miRNA-gene validated relations, from Diana Tarbase and/or miRecords, provided an independent true solution for comparative evaluation. The figure shows the variation of “enrichment score” (ratio between the observed number of validated relations, included in the selected set of supported relations, and the expected number of validated relations, based on proportions) against stringency of anti-correlation-based percentile cutoff. Each dataset is considered separately to compare REAL and PROXY analysis methods. The REAL method is able to enrich in validated relations, outperforming the PROXY, when it focuses on anti-correlated miRNA-target subsets defined with high stringency. Also the REAL method looses any power, as expected, at low stringency.

A set of 2, 848 validated miRNA-target interactions, resulting from Diana Tarbase [33] and/or miRecords [34] was collected to provide an independent, also if narrow, true solution for comparative evaluation. In total, 756 validated miRNA-target relations were represented in the considered set of predicted relations, with different small subsets represented for different expression datasets. The average of total numbers of predicted relations associated to negative correlation values in different datasets (representing the group from which we selected most supported relations according to anti-correlation ranking cutoffs) was about 81, 500. For each dataset and each threshold, we evaluated the number of validated relations included in the selected set of supported relations, according to the Real and the Proxy analysis, as compared with the expected number of validated relations. The ratio between observed and expected numbers of validated relations included in a selected set of supported relations defines an “enrichment score”, measuring the helpfulness of expression profiles anti-correlation analysis to identify functional regulatory interactions among simply predicted relations. Figure 4 reports the variation of enrichment score, against stringency of anti-correlation-based percentile threshold, for each considered expression dataset. Plainly, the REAL analysis is able to enrich in validated relations, when it focuses on anti-correlated miRNA-target subsets defined with high stringency (from 1% to 5%), but looses its power, as expected, at lower stringency. Besides, the REAL analysis results outperform those of the PROXY, which seems to find, almost in all datasets, proportions of validated (over supported) relations comparable or even lower than expected by chance, almost independently from the applied stringency on anti-correlation. We observed also that, for each considered expression dataset, the groups of validated relations detected by the REAL and PROXY methods are almost completely disjointed.

## Discussion

In this study we exploited sequence, genomic and expression information to investigate two main aspects of post-transcriptional regulation in miRNA biogenesis, namely strand selection and expression relationships between intragenic miRNAs and host genes.

Our observations were based on a comprehensive collection of miRNAs and genes/transcripts whose annotation and localization was integrated with expression profiles computed from five large microarray-based datasets, regarding different biological contexts and including both normal and tumour/disease samples. At least 8 different tissues types are represented (breast, prostate, liver, ovary, testes, lung, colon and brain) plus different T- and B-lineage blood cells. The high number of samples and the broad coverage of cell types would guarantee both significance and fair generality of the obtained results.

The first evidence emerging from our analysis regards the expression behaviour of pairs of sister mature miRNAs produced from the same hairpin. As mentioned above, in the classic model of miRNA biogenesis, the duplex of mature miRNAs is produced by Dicer processing of the hairpin precursor. Then, a following strand selection step determines which mature miRNA is the degraded “passenger” strand and which is the major and stable form that will act as guide for the mature miRISC complex in the post-transcriptional silencing of target genes. It is worth notice that the two mature miRNAs have different sets of target genes and may differently contribute to the regulation of cell activities. Our analysis of expression profiles showed that, considering different samples, representing different tissue types under various conditions, the strand selection is highly regulated. In fact, we observed miRNA pairs in which the same miRNA is the most expressed in the majority of considered samples, as well as miRNA pairs expressed at comparable level in almost all considered samples. Nevertheless, the large majority of miRNA pairs show a not deterministic strand selection bias, which may be highly regulated since it shows tissue-/cell-/condition-specific modulation. This is confirmed by the fact that unsupervised analysis, using for samples classification only information about the direction and the strength of the strand selection bias, is able to distinguish different tissues, and sometimes also different conditions (as normal and malignant cells). Moreover, when considering a minimum expression threshold, only a minority of pairs were expressed alternatively in all considered cell types, whereas the majority were concurrently expressed in some cell types and alternatively in others. A significant fraction of concurrently expressed pairs showed highly comparable levels, suggesting that both the major and the minor forms may contribute to post-transcriptional gene silencing, possibly in a coordinate way. Also considering separately alternatively and concurrently expressed pairs, the regulated nature of the strand selection is evident. Our results on genome-wide investigation of co-expression relationships of sister miRNAs showed that different biological contexts share complex mechanisms of strand selection regulation. This observation complements recent findings coming from deep sequencing of short RNAs which recently conducted to identification of new miRNAs [35], characterisation of miRNAs variants (isomiRs; partly produced by alternative processing of precursors) [36], [37], and discovery of a novel class of miRNA-related RNA (micro-RNA offset RNAs; moRNAs) distinct from miRNAs, but derived from miRNAs precursors [38]. Indeed, the complexity of miRNA biogenesis regulation is indicated by emerging evidences coming from the present study of miRNAs expression and from sequencing data analysis, continuously adding novel layers to miRNAs biogenesis pathways and enriching possibilities for their regulation.

The second aim of the study was to clarify to what extent intragenic miRNAs were co-expressed with the corresponding host genes. An intragenic miRNAs and host genes related behaviour has been taken for proven by different Authors and used as a strong assumption for the design of computational methods for miRNA targets identification [26] or to go further and explore the possible role of intragenic miRNAs in supporting the regulatory activity of host genes products [39]. We considered four expression datasets including expression profiles in various cell types (brain in normal and with Alzheimer disease conditions, normal prostate and prostate cancer, normal blood cells and different blood cell diseases) and clearly showed, that the large majority of intragenic miRNAs do not share similar expression profiles with their host genes. Only 10% of miRNA and host gene pairs appear significantly co-expressed. This may be partially explained by the fact that not all miRNAs located in introns of protein coding genes are under the transcriptional control of coding gene promoter(s). In fact, Corcoran and colleagues (2009) [40] experimentally identified mammalian miRNA Polymerase II promoters by chromatin immunoprecipitation. They discovered that the nearest ChiP-chip peak for a number of intragenic miRNAs overlaps the host gene's TSS but that reportedly one quarter of intragenic miRNAs may be transcribed from their own promoters and thus showing different expression behaviour and modulation than the protein-coding gene transcript(s). This result, as well our findings and considerations, encourage much more detailed studies about transcriptional regulation of miRNAs expression.

Results of the last part of our study are relevant mainly from a technical and methodological point of view. As previously said, host genes expression profiles were proposed as possible proxies for the intragenic miRNA expression profile, when the latter is unavailable, to identify most probable miRNA target genes. We conducted two integrated analysis of target prediction and expression profiles, one using real miRNA and gene expression profiles, and the second using host genes expression profiles as proxy for miRNAs, and gene expression profiles. The comparative evaluation of the two methods was based on an independent true solution, represented by a set of validated regulatory interactions. This allowed to measure and compare the effectiveness of the two methods in finding validated regulatory interactions, among the subset of predicted miRNA-target relations supported by negative correlations of expression profiles. Our results support the usefulness of the integrated analysis conducted on real miRNAs expression profiles, when stringency is kept reasonably high. Moreover, as expected from previous observations about intragenic miRNAs and host genes scarce co-expression, we experienced that the use of host genes expression as a proxy for miRNA profiles for the integrated analysis seems not significantly enrich in validated relations.

In conclusion, our analysis of miRNA sister pairs expression prevalence across samples from five sizeable and diverse expression datasets showed that the large majority of miRNA pairs show a not deterministic strand selection bias, which may be highly regulated, since it presents tissue-/cell-/condition-specific modulation. Our conclusions strengthened recent evidences about the previously underestimated importance of the strand selection regulation, reinforcing the role of such layer of miRNA biogenesis in miRNA-based control of cell activities. Furthermore, our results showed that most host genes and intragenic miRNAs are scarcely co-expressed. In specific cases, they might be co/expressed but mainly in a cell/tissue-specific way. This actually does not rule out the importance of already documented cooperation of specific intragenic miRNAs and host genes products, but proves that the expression information of corresponding host genes can hardly be used as estimator for actual expression of the co-transcribed miRNA and encourage more detailed studies of transcriptional regulation of miRNAs expression.

## Materials and Methods

### microRNAs and genes: sequences and genomic positions

We obtained 49, 506 human genes and 132, 056 transcripts sequences from ENSEMBL (version 56) each associated to a unique chromosomal position. The complete set of hairpin precursors of human microRNA sequences was downloaded from miRBase version 14, thus obtaining a set of 721 pre-miRNA hairpin sequences and 904 mature miRNAs, 185 of which are tagged as “minor”, according to miRBase annotation (i.e. hsa-miR-30e*). Hairpin miRNA sequences were aligned with the version 37.1 of the human genome to establish their genomic positions as start and end coordinates of the aligned region in a specific chromosome and strand. Alignments associated to at least 95% sequence identities, calculated over the hairpin sequence length, have been considered for miRNA genome position definition. As genomic localization is referred to hairpin sequences whereas miRNA microarray platforms measure expression profiles of mature miRNAs, mature miRNAs to hairpin correspondence info was used for data integration. miRNA hairpins localizations were compared with those of protein-coding genes to identify intragenic miRNAs, putatively transcribed from the coding gene promoter [see Table S1 for miRNA annotation, genomic localization and corresponding host gene]. To define the miRNA-host gene relationships considered in further analyses, only miRNAs fully included in genes spanned regions were considered as intragenic. Specifically, 367 miRNAs were categorized as intergenic and thus excluded, whereas 309 intragenic miRNAs, were associated with 279 protein-coding human host genes. Among these, 23 (8.5%) include at least two miRNAs.

### Expression datasets and chips annotation

#### Multiple Myeloma dataset (MM)

MM dataset consists of matched miRNAs and genes expression profiles from purified plasma cells of thirty-nine human samples, including 33 patients with multiple myeloma (MM), 2 with plasma cell leukaemia (PCL) and 4 healthy donors. The miRNA expression was profiled on the Agilent Human miRNA Microarray V2. The human miRNAs data were re-annotated and normalized as suggested in [20]. MiRNA data are publicly available under GEO accession GSE17498. The gene expression was profiled on Affymetrix GeneChip® Human Gene 1.0 ST Array. The raw intensity signals of genes were extracted from CEL files and normalized using the default settings of *affy* package for Bioconductor and re-annotated using Manhong Dai custom cdf, HuGene10stv1_Hs_ENSG (available at http://brainarray.mbni.med.umich.edu/Brainarray/Database/CustomCDF/12.1.0/ensg.asp).

#### Acute Lymphoblastic Leukaemia dataset (ALL)

ALL dataset consists of matched miRNA and genes expression profiles in nineteen adult Acute Lymphoblastic Leukaemia (ALL) cases, including T-lineage and B-lineage cells, harbouring specific molecular lesions [28](GEO accession GSE14834). Human miRNA data obtained by Lc Sciences Human 470 miRHuman 9.0 microarray were processed using the same approach suggested by the original paper. Gene expression was profiled on Affymetrix GeneChip® Human Genome U133 Plus 2.0 Array. The raw intensity signals of genes were extracted from CEL files and normalized using the default settings of affy package for Bioconductor and re-annotated using Manhong Dai custom cdf, HGU133Plus2_Hs_ENSG (available at http://brainarray.mbni.med.umich.edu/Brainarray/Database/CustomCDF/12.1.0/ensg.asp).

#### Normal and Alzheimer's parietal lobe cortex (ALZ)

ALZ dataset consists of 16 matched miRNA and gene expression experiments, obtained by USC/XJZ Human 0.9 K miRNA-940-v1.0 and Affymetrix Human Genome U133 Plus 2.0 Array, in parietal lobe tissue from 4 Alzheimer Disease patients and 4 age-matched controls (GSE16759) [41]. Human miRNA data were processed using the same approach suggested by the authors. Gene expression was profiled on Affymetrix GeneChip® Human Genome U133 Plus 2.0 Array. The raw intensity signals of genes were extracted from CEL files, normalized using the default settings of affy package for Bioconductor, and re-annotated using Manhong Dai custom cdf, HGU133Plus2_Hs_ENSG.

#### Normal prostate and prostate cancer (PRO)

PRO dataset consists of the subset of 140 matched miRNA and gene expression experiments, obtained respectively by Agilent-019118 Human miRNA Microarray 2.0 and Affymetrix Human Exon 1.0 ST, of the prostate data reported in [42](GEO accession GSE21032) regarding primary and metastatic prostate cancer samples and control normal adjacent benign prostate. Human miRNA data were processed using the same approach suggested by the original paper. Gene expression profiles was obtain using RMAExpress, a standalone GUI program to compute gene expression summary values for Affymetrix Genechip data using the Robust Multichip Average expression summary and to carry out quality assessment using probe-level metrics.

#### Multiple cancers and normal tissues dataset (MCN)

MCN dataset includes miRNAs expression profiles in 32 samples from 14 different patients and 8 different tumour types, with tumour cells and normal cells counterpart for each patient [29](GEO accession GSE14985). Tissue samples were from various embryonic lineages: one pair from breast, lymphoma and prostate; two from liver, ovary, testes and lung and three from colon: two technical replicates are included for ovary and testes samples. MiRNA expression was profiled using Agilent Human miRNA Microarray 2.0, and the data processed using the same approach suggested by Navon et al.

### Analysis of sister miRNA pairs expression ratio

Mature miRNA pairs may be generated by both 5′ and 3′ strands of the hairpin stem. For historical reasons, miRNA names are assigned in miRBase according to an inhomogeneous notation. miR-X/miR-X* sister pairs refers to the fact that one miRNA of the pair, the miRNA*, is the minor form. An older convention used miR-142-s and miR-142-as. For other pairs, the strand is explicitly indicated in the mature miRNA name (miR-X-5p/miR-X-3p). The latter naming convention is most informative and unambiguous and it will be used for future miRBase releases. Thus, in the present study, we report mature miRNA according to current miRBase names but the order of mature miRNAs in considered sister pairs was kept consistent with the physical position of mature sequences in the precursor, i.e. miRNAs are given in 5′ to 3′ order. For each sister miRNA pair represented in at least one of the five considered datasets, we calculated the per sample log_2_(ratio) between expression values of 5′ and 3′ sister miRNAs. Matrix values were standardised and used for cluster analysis of samples and of miRNA pairs, using Euclidean distance and average clustering. Then, for each dataset we considered not expressed in a given sample those miRNAs associated to expression values lower than the median of the dataset expression matrix (i.e. low values were set to 0). We calculated the per sample log_2_(ratio) between expression values of sister miRNAs as indicated before, but miRNA pairs expressed in alternative way in a given sample were associated to extreme values. When only one out of two sister miRNAs was expressed over the threshold, log_2_(ratio) values (generating ±∞) were artificially set to maxLog(ratio)+0.1, if only the 5′ miR is expressed, or to minLog(ratio)-0.1 in the opposite case. Values of log_2_(ratio) of samples in which both miRNAs of the pair are not expressed were not considered for the clustering analyses.

### Target predictions and integrated analysis of miRNAs and target genes expression profiles

The integrated analysis of miRNAs and target genes expression profiles combines target predictions with miRNAs and gene expression data correlation-based analysis to identify, among predicted target genes for each considered miRNA, those regulatory relationships significantly supported by expression data.

In details, the procedure comprises the identification of miRNA target genes by computational predictions and compilation of the adjacency matrix of targeting relationships, and the computation of pair-wise relatedness of miRNAs and targets from matched expression matrices, to identify relationships supported by expression data, which could be used for post-transcriptional regulatory networks reconstruction and study. The set of microRNA-target predictions were defined using TargetScan 5.1 considering both conserved and non conserved sites that match the seed region of each miRNA. The pair-wise Pearson correlation coefficient between miRNA and target genes expression profiles in exactly the same samples was calculated. We then selected as reliable and potentially functional the subset of predicted relationships associated to most negative Pearson coefficients. Different percentile-based cutoffs were applied, to define the groups of supported regulatory interactions.

## Supporting Information

Figure S1 **Variability of strand selection bias across samples among all considered datasets.** Figure S1 shows patterns prevalence for a set of 5 5′/3′ sister miRNA pairs obtained by the combination of all considered datasets.
(TIF)

Figure S2 **Variability of strand selection bias across samples considering miRNA pair classification.** Figure S2 reports patterns prevalence for a set of 95 5′/3′ sister miRNA pairs obtained by the combination of three out of five considered datasets giving rise to the maximum number of represented miRNA pairs (MM, PRO and MCN). As detailed in Methods, miRNA pairs concurrently or alternatively expressed were associated respectively to the per sample standardised log_2_(ratio) and to extreme values derived from observed distribution. Cluster analysis performed with these values, produce an heatmap showing both the regulation of the strand selection bias and alternative expression occurrence in different samples.
(TIF)

Table S1 **miRNAs genomic localization.** Table S1 includes miRNAs genomic localization and host genes.
(TXT)
